# Assessment of the Cepheid 3-gene Host Response Fingerstick Blood Test (MTB-HR) on rapid diagnosis of tuberculosis

**DOI:** 10.1080/22221751.2023.2261561

**Published:** 2023-10-17

**Authors:** Xiaocui Wu, Guangkun Tan, Jian Ma, Juan Yang, Yinjuan Guo, Haiwen Lu, Hui Ke, Mengran Li, Yi-Wei Tang, Wei Sha, Fangyou Yu

**Affiliations:** a Department of Clinical Laboratory, Shanghai Pulmonary Hospital, School of Medicine, Tongji University, Shanghai, People’s Republic of China; b Shanghai Key Laboratory of Tuberculosis, Shanghai Pulmonary Hospital, Tongji University School of Medicine, Shanghai, People’s Republic of China; c Department of Clinical Laboratory, Shanghai University of Traditional Chinese Medical Attached Shuguang Hospital, Shanghai, People’s Republic of China; d Medical Affairs, Danaher Diagnostic Platform People’s Republic of China/Cepheid, Shanghai, People’s Republic of China; e Department of Tuberculosis, Shanghai Pulmonary Hospital, School of Medicine, Tongji University, Shanghai, People’s Republic of China; f Department of Respiratory and Critical Care Medicine, Shanghai Pulmonary Hospital, School of Medicine, Tongji University, Shanghai, People’s Republic of China; g Department of Biostatistics & Data Management, Beckman Coulter People’s Republic of China, Danaher, Shanghai, People’s Republic of China; h Department of Laboratory Medicine, The First Affiliated Hospital of Wenzhou Medical University, Wenzhou, People’s Republic of China

**Keywords:** Cepheid 3-gene Host Response Fingerstick Blood Test, active tuberculosis, latent *Mycobacterium tuberculosis* infection, other pulmonary diseases, healthy volunteers

## Abstract

The World Health Organization has identified high-priority target product profiles for new TB diagnostics which include rapid biomarker-based, non-sputum-based diagnostic testing, using an easily accessible sample. The Cepheid 3-gene Host Response Fingerstick Blood Prototype Test (MTB-HR) quantifies relative mRNA levels of a 3-gene signature (*GBP5*, *DUSP3*, and *KLF2*) from a whole-blood sample on the GeneXpert platform. The objective of the present study was to evaluate the performance of the MTB-HR to distinguish between active tuberculosis (ATB), latent *Mycobacterium tuberculosis* infection (LTBI), other pulmonary diseases, and healthy volunteers at a tertiary care centre. Among 653 participants enrolled in this study, 192 were diagnosed as having ATB, and the remaining 461 were classified as non-ATB, including 137 cases of LTBI, 224 cases of other pulmonary diseases, and 100 healthy volunteers. The corresponding AUCs of the MTB-HR in distinguishing untreated ATB from non-ATB, LTBI, other pulmonary diseases, and healthy volunteers were 0.814 (95% CI, 0.760-0.868, sensitivity 76.1%, specificity 71.6%), 0.739 (95% CI, 0.667-0.812, sensitivity 59.7%, specificity 78.1%), 0.825 (95% CI, 0.770-0.880, sensitivity 82.1%, specificity 65.6%), 0.892 (95% CI, 0.839-0.945, sensitivity 76.1%, specificity 88.0%), respectively. When only samples with TAT of less than 1 h were included, the AUC of the MTB-HR in distinguishing untreated ATB from non-ATB was largest, 0.920 (95% CI, 0.822-1.000, sensitivity 81.3%, specificity 87.7%). In conclusion, the MTB-HR assay shows potential as a rapid, blood-based screening and triage test for ATB, especially for untreated ATB, with the advantage of increased diagnostic yield since blood is more readily available.

## Introduction

To achieve the goal of the “End TB strategy,” timely and accurate detection of *Mycobacterium tuberculosis* is essential for the treatment and prevention of TB. At present, the laboratory diagnosis of tuberculosis mainly relies on smear, culture and molecular biology techniques and the testing of sputum or samples obtained by invasive sampling. Interferon gamma release assays (IGRAs) are in vitro diagnostic assays that assist in the diagnosis of tuberculosis by detecting the release of specific IFN-γ in blood samples. However, IGRAs have limitations. Infection caused by *Mycobacterium kansasii*, *Mycobacterium szulgai* and *Mycobacterium gordonae* has the potential to cause false positive results. As well, IGRAs are not recommended for detecting active TB in all settings [1]. The World Health Organization’s (WHO) high-priority target product profiles for new TB diagnostics reported in 2014, included a rapid, biomarker-based, non-sputum-based diagnostic test to accurately diagnose TB using an easily accessible sample [2]. Currently, blood-based TB biomarkers are mainly focused on host immune response markers, including metabolic, proteomic and transcriptomic signatures of disease [3]. A multi-cohort study by Sweeny et al showed that a group of three genes (*GBP5*, *DUSP3*, and *KLF2*) were highly diagnostic for ATB. The area under the receiver operating characteristic curve (ROC) of the 3-gene signature in distinguishing ATB from healthy controls, LTBI and other diseases was 0.90 (95% CI, 0.85–0.95, sensitivity 85%, specificity 93%), 0.88 (95% CI, 0.84–0.92, sensitivity 80%, specificity 86%) and 0.84 (95% CI, 0.80–0.95, sensitivity 81%, specificity 74%), respectively [4]. Subsequently, they conducted a prospective study to assess the effectiveness of the three genes score for TB progression and diagnosis, disease severity and treatment response [5]. This study found the three genes score was associated with progression from LTBI to ATB 6 months prior to sputum conversion with 86% sensitivity and 84% specificity, diagnosed patients with ATB with 90% sensitivity and 70% specificity, and correlated with treatment response and the severity of lung pathology [5].

Cepheid (Sunnyvale, CA, USA) has developed a 3-gene Host Response Fingerstick Blood Prototype Test (MTB-HR) that quantifies relative mRNA levels of the 3-gene signature from a whole-blood sample on the GeneXpert platform. Currently, a limited number of studies have been performed evaluating the clinical diagnostic value of the MTB-HR, specifically in a prison population and persons living with HIV [6, 7]. The interim data from a prospective, multi-site study from patients presenting with symptoms suggestive of TB but prior to microbiological confirmation showed that the MTB-HR reached the minimal target product profile for a point of care triage test for TB regardless of geographic area or HIV infection status [8]. The aim of this study was to evaluate the performance of the MTB-HR among persons with ATB, LTBI, other pulmonary diseases, and healthy volunteers at a tertiary care centre in China.

## Materials and methods

### Study design and participants

This prospective study was conducted in Shanghai Pulmonary Hospital (SPH), Tongji University School of Medicine. SPH is a tertiary-care hospital which specializes in the care of persons with pulmonary diseases and is also a national designated tuberculosis hospital. Patients enrolled in the study were inpatients from April 16, 2020 to July 14, 2020. The criteria for inclusion were persons over 12 years of age and those who tested HIV-negative prior to enrollment. Demographic information, including sex, age, medical history, and underlying conditions, were recorded upon enrollment. IGRA, sputum or lavage fluid microbiological testing and follow-up including clinical diagnosis were recorded, as well. Further information on TB exposure history, treatment history within 6 months, and clinical symptoms were collected for patients who were ultimately diagnosed with ATB. This study was approved by the Ethical Committee of SPH.

Patients were classified into three cohorts in this analysis: (i) ATB, (ii) LTBI, and (iii) other pulmonary diseases. Using a comprehensive microbiological reference standard (CMRS), The ATB cohort comprised individuals who exhibited radiographic abnormalities and had microbiological evidence of tuberculosis in their sputum or lavage fluid, as confirmed by microbiological analysis (culture or Xpert MTB/RIF [Cepheid, Sunnyvale, USA]). Additionally, they had a clinical diagnosis of confirmed tuberculosis. LTBI was defined as asymptomatic individuals with a positive IGRA result, a normal chest radiograph, no clinical symptoms of TB, and no development of ATB within 6 months. Other pulmonary diseases were defined as other pulmonary diseases without a history of TB exposure and a negative or uncertain IGRA result. LTBI participants were excluded if they developed tuberculosis disease within 6 months of enrolment to exclude early asymptomatic disease that could have been present at the time of assessment. In addition, we set up a healthy volunteer group which included people who received normal results from a routine physical examination at SPH on November 5, 2020.

Laboratory testing (smear, culture, IGRA, Xpert MTB/RIF, the MTB-HR) were performed at the clinical laboratory, an ISO 15189 accredited laboratory specialized in MTB detection. All laboratory examinations were conducted by professional staff in accordance with standard operating procedures. The smears were examined using fluorescence staining and a Bactec MGIT 960 instrument (Becton Dickinson, Cockeysville, MD, USA) was used for culture. All MTB isolates were confirmed using MBP 64 antigen detection kits (Genesis, Hangzhou, China).

The MTB-HR was performed according to the manufacturer’s instructions. Blood samples were taken from the whole blood obtained for routine blood testing. The specimens were placed at 4°C and tested within 24 h. A sample containing 100 uL of blood was directly transferred into the MTB-HR and subsequently analyzed using a GeneXpert instrument. The MTB HR test time was 50 min and a 3-gene TB score was calculated from threshold cycle (CT) values: 3-gene TB score = ([CT (GBP5) + CT (DUSP3)]/2) − CT(KLF2) [4]. The turnaround time (TAT) from collection to detection of the MTB-HR is collected.

### Statistical analysis

Mean, medium, range, IQR (Interquartile range), variance and standard deviation of TB score in different population were calculated. The diagnostic performance of the obtained TB score was evaluated by ROC (receiver operating characteristic) curve analysis using the diagnostic categories definite ATB and non-ATB / LTBI / other pulmonary diseases / healthy volunteers as binary reference standards for the classifier. The diagnostic performance of the obtained TB score was also evaluated by ROC curve analysis using the diagnostic categories definite ATB and non-ATB / LTBI / other pulmonary diseases / healthy volunteers as binary reference standards for the classifier. For ATB versus non-ATB and naive ATB versus non-ATB, the optimal cut off value was determined by maximizing the Youden index. Meanwhile, sensitivity and specificity were calculated at the threshold value that maximized the Youden index. To evaluate if the test fulfills the WHO requirements for a triage test, specificity was also calculated at the threshold value corresponding to 90% sensitivity (minimal target). In addition, pairwise comparisons of the TB score mean in different groups were performed using Tukey’s multiple comparison test. Medcalc software, Prism 9 and SAS version 9.4 were used for data analysis and figure production.

## Results

### Clinical characteristics

A total of 653 participants were enrolled in this study. 192 were diagnosed as ATB, and the remaining 461 were non-ATB, including 137 cases of LTBI, 224 cases of other pulmonary diseases, and 100 healthy volunteers. Other pulmonary diseases included sarcoidosis, pulmonary infection, interstitial pulmonary disease, and lung tumours. Patients with ATB were classified as into one of 3 groups; 67 patients with ATB who had not received treatment (called “naive ATB”), 29 patients who received anti-TB treatment duration between 0 and 8 weeks (called “ATB 0-8w”), and 96 patients who received anti-TB treatment duration longer than 8 weeks (called “ATB ≥ 8w”). Of the 192 ATB cases, 149 were culture-positive, 102 were smear-positive and 159 were Xpert MTB/RIF-positive.

The demographic characteristics of the included participants are shown in Table 1. Among them, 425 (65.1%) were males. Their ages ranged from 15 to 85 years old. Of the 653 patients, 304 had positive IGRA results, 231 had negative IGRA results, and the remaining 18 had inconclusive results. Of the 653 patients, 320 had all three pair matched comparisons of smear, culture and Xpert MTB/RIF results. Of the 192 cases of ATB, 1 had a history of tuberculosis exposure, 18 had a previous history of tuberculosis, and 34 had typical clinical symptoms of tuberculosis. The TAT varies from 0 to 24 h. Of these, 99 (15.2%) were tested for the MTB-HR within 1 h after blood collection. The epidemiological risk of ATB, LTBI and other pulmonary diseases included in the study are shown in Table 2. Among the 553 patients, 76 (13.7%) had hypertension, 48 (8.7%) had diabetes, 84 (15.2%) had a history of smoking, and 14 (2.5%) had a history of alcohol consumption.

**Table 1.** Demographic characteristics in this study (*n* = 653).

		Total	ATB	LTBI	Other pulmonary diseases	Healthy volunteer	Non-ATB total
Sex	Female	228 (34.9%)	74 (38.5%)	48 (35%)	89 (39.7%)	17 (17%)	154 (33.4%)
	Male	425 (65.1%)	118 (61.5%)	89 (65%)	135 (60.3%)	83 (83%)	307 (66.6%)
Age	Median (IQR)	53 (34, 65)	37 (26, 60)	59 (41, 69)	62 (51, 69)	38 (31, 48)	55 (40, 67)
	≤20	22 (3.4%)	14 (7.3%)	7 (5.1%)	1 (0.4%)		8 (1.7%)
	20–40	204(31.2%)	92 (47.9%)	27 (19.7%)	23 (10.3%)	62 (62%)	112(24.3%)
	40–60	197 (30.2%)	41 (21.4%)	39 (28.5%)	80 (35.7%)	37 (37%)	156 (33.8%)
	>60	230(35.2%)	45 (23.4%)	64 (46.7%)	120 (53.6%)	1 (1%)	185 (40.1%)
IGRA	Negative	231 (41.8%)	17 (8.9%)		214 (95.5%)		214 (59.3%)
	Positive	304 (55%)	167 (87%)	137 (100%)			137 (38%)
	Uncertain	18 (3.3%)	8 (4.2%)		10 (4.5%)		10 (2.8%)
Tuberculosis exposure history	Yes	1 (0.4%)	1 (0.5%)				
	No	273 (99.6%)	191 (99.5%)	66 (100%)	16 (100%)		82 (100%)
History of TB	Yes	31 (11.3%)	18 (9.4%)	11 (16.7%)	2 (11.8%)		13 (15.7%)
	No	244 (88.7%)	174 (90.6%)	55 (83.3%)	15 (88.2%)		70 (84.3%)
^a^Typical clinical symptoms of tuberculosis	Yes	41 (14.9%)	34 (17.7%)	6 (9.1%)	1 (5.9%)		7 (8.4%)
	No	234 (85.1%)	158 (82.3%)	60 (90.9%)	16 (94.1%)		76 (91.6%)
TAT	0–1	99 (15.2%)	26 (13.5%)	22(16.1%)	26 (11.6%)	25 (25.0%)	73 (15.8%)
	1–2	99 (15.2%)	23 (12.0%)	23 (16.8%)	48 (21.4%)	5 (5.0%)	76 (16.5%)
	2–3	98 (15.0%)	20 (10.4%)	15 (10.9%)	31 (13.8%)	32 (32.0%)	78 (16.9%)
	3–4	95 (14.5%)	28 (14.6%)	15 (10.9%)	24 (10.7%)	28 (28.0%)	67 (14.5%)
	4–5	51 (7.8%)	6 (3.1%)	12 (8.8%)	23 (10.3%)	10 (10.0%)	45 (9.8%)
	5–6	29 (4.4%)	11 (5.7%)	8 (5.8%)	10 (4.5%)	0 (0.0%)	18 (3.9%)
	>6	182 (27.9%)	78 (40.6%)	42 (30.7%)	62 (27.7%)	0 (0.0%)	104 (22.6%)

^a^ Typical clinical symptoms of tuberculosis include low fever after noon, fatigue, loss of appetite, weight loss, and night sweats.

*The turnaround time (TAT) from collection to detection of the MTB-HR is collected. TAT is calculated in hours.

**Table 2.** The epidemiological risk of ATB, LTBI and other pulmonary diseases included in the study.

Epidemiologic risk	Total	ATB	LTBI	Other pulmonary diseases
Hypertension	76 (13.7%)	14 (1.3%)	10 (7.3%)	52 (23.2%)
Diabetes	48 (8.7%)	14 (1.3%)	14 (10.2%)	20 (8.9%)
Coronary heart disease	9 (1.6%)	1 (0.5%)	4 (2.9%)	4 (1.8%)
Smoke	84 (15.2%)	13 (6.8%)	29 (21.2%)	42 (18.8%)
Alcohol consumption	14 (2.5%)	3 (1.6%)	5 (3.7%)	6 (2.7%)
Dust exposure history	8 (1.5%)	0 (0%)	3 (2.2%)	5 (2.2%)

The microbiological examination results of sputum or bronchoalveolar lavage fluid showed that 25 cases of ATB were positive on microbial culture for other bacteria. Among them, there were 11 cases of *Klebsiella pneumoniae*, 3 cases of *Stenotrophomonas maltophilia*, 2 cases of *Escherichia coli*, 2 cases of *Serratia marcescens*, 2 cases of *Acinetobacter baumannii*, 2 cases of *Enterobacter cloacae complex*, 2 cases of *Nontuberculous mycobacteria* (NTM), 1 case of *Pseudomonas aeruginosa*, and 1 case of *Achromobacter xylosoxidans*. In addition, there was one case where *Klebsiella pneumoniae* and *Acinetobacter baumannii* were detected simultaneously, and another case where *Klebsiella pneumoniae* and *Aspergillus* were detected simultaneously. Of the 461 non-ATB patients, 89 cases had bacterial infections as evidenced by a positive culture on sputum or bronchoalveolar lavage fluid and 17 cases had fungal infections. Among the bacterial infections, NTM was the most common (35 cases), followed by *Klebsiella pneumoniae* (13 cases) and *Pseudomonas aeruginosa* (9 cases). Among the fungal infections, *Aspergillus* was the most common with 7 cases.

### Evaluation of 3-gene TB score in participants from different cohorts using CMRS

**Figure 1.**
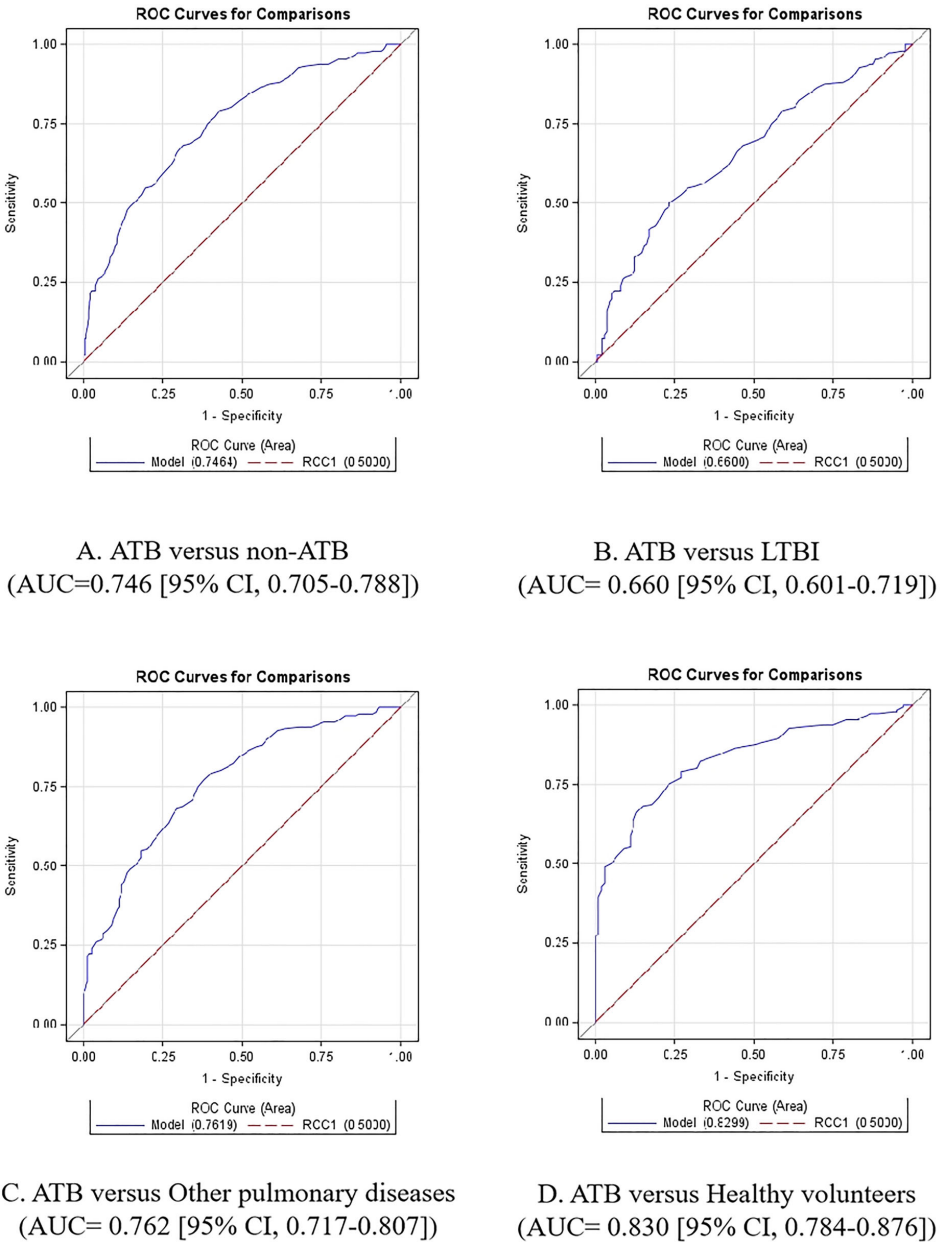
ROC curves for distinguishing ATB from non-ATB, latent tuberculosis, other pulmonary diseases, and healthy volunteers.

The mean 3-gene TB scores of the MTB-HR in ATB, LTBI, other pulmonary diseases, and healthy volunteers were 1.815 (95% CI, 1.665-1.965), 2.380 (95% CI, 2.228-2.531), 2.768 (95% CI, 2.658-2.877) and 2.934 (95% CI, 2.832-3.036), respectively. We compared the 3-gene TB score in participants from the various cohorts (Figure 1). As expected, the 3-gene TB score distinguished ATB from non-ATB (AUC of 0.746 [95% CI, 0.705–0.788]), latent tuberculosis (AUC of 0.660 [95% CI, 0.601–0.719]), other pulmonary diseases (AUC of 0.762 [95% CI, 0.717–0.807]), and healthy volunteers (AUC of 0.830 [95% CI, 0.784–0.876]). Optimal sensitivity and specificity were obtained for the MTB-HR when the Youden index was maximized. For ATB versus non-ATB, the cut-point sensitivity was 66.1% [95% CI, 59.2–72.5], and the specificity was 70.3% [95% CI, 66.0–74.3]. Considering Xpert-MTB-HR as a triage test, at a sensitivity of 90%, specificity was 35.4% [95% CI, 31.0–39.9].

**Figure 2.**
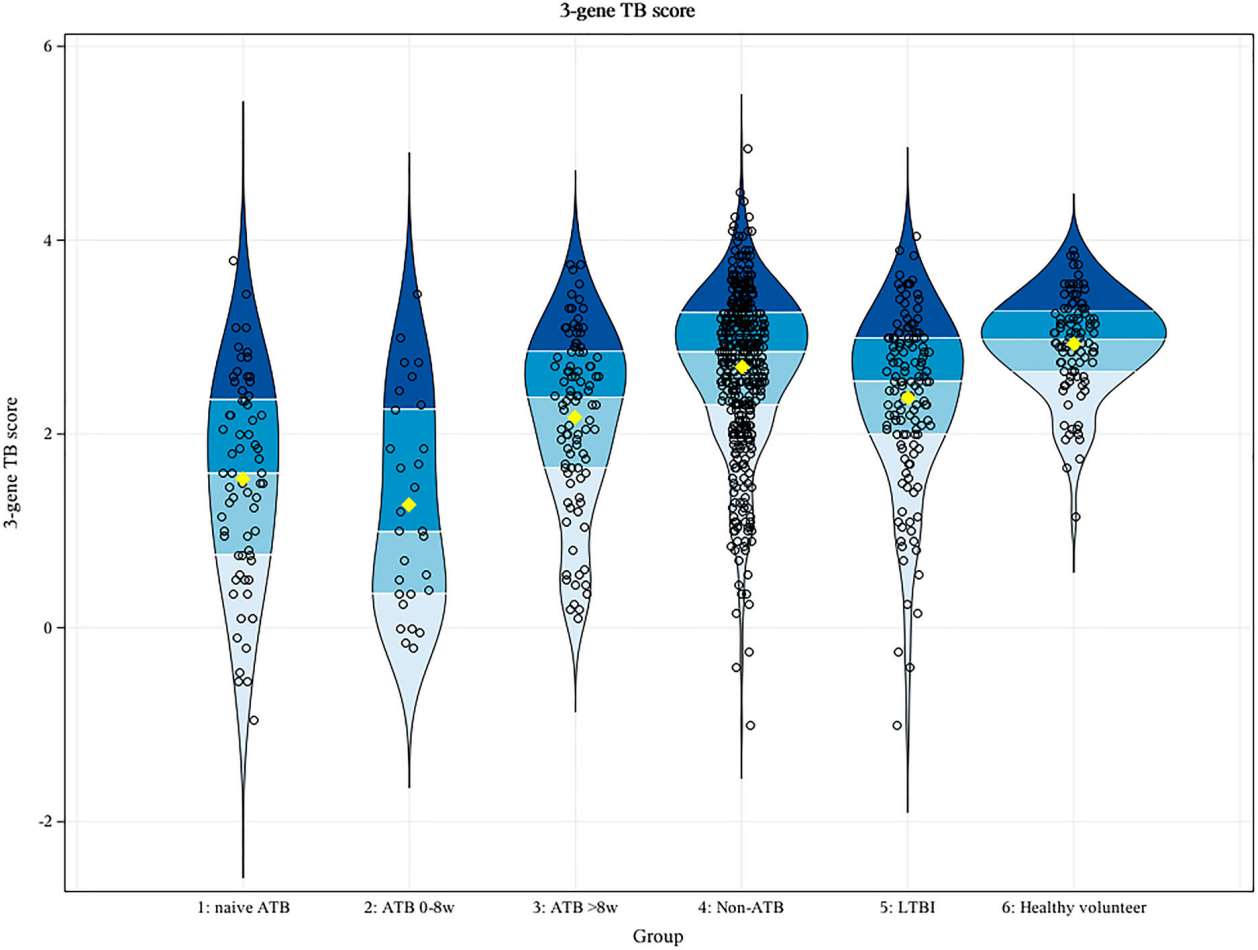
Comparison of 3-gene TB score in participants with ATB without treatment, different duration of previous anti-TB treatment, non-ATB, latent tuberculosis and healthy volunteers. The outer shape of the violin plot represents all possible results. The next layer inside represents the values that occur 50% of the time. Yellow square: mean 3-gene TB score (naive ATB: 1.530; ATB 0-8w: 1.272; ATB >8w: 2.178; Non-ATB: 2.688; LTBI: 2.380; Healthy volunteer: 2.934).

Figure 2 showed the comparison of 3-gene TB score in participants with ATB without treatment, different duration of previous anti-TB treatment, non-ATB, latent tuberculosis and healthy volunteers. The mean 3-gene TB scores of the MTB-HR in naive ATB, ATB 0-8w, ATB ≥ 8w, non-ATB, latent tuberculosis, and healthy volunteers were 1.530 (95% CI, 1.272–1.788), 1.272 (95% CI, 0.862–1.683), 2.178 (95% CI, 1.993–2.364), 2.688 (95% CI, 2.613–2.764), 2.380 (95% CI, 2.228–2.531) and 2.934 (95% CI, 2.832–3.036) respectively. Tukey’s multiple comparisons showed that pair comparisons of naive ATB, ATB 0-8w, ATB ≥ 8w, and non-ATB showed the *p* values were less than 0.0001, except for the *p* value of 0.549 in the comparison of naive ATB and ATB 0-8w.

**Figure 3.**
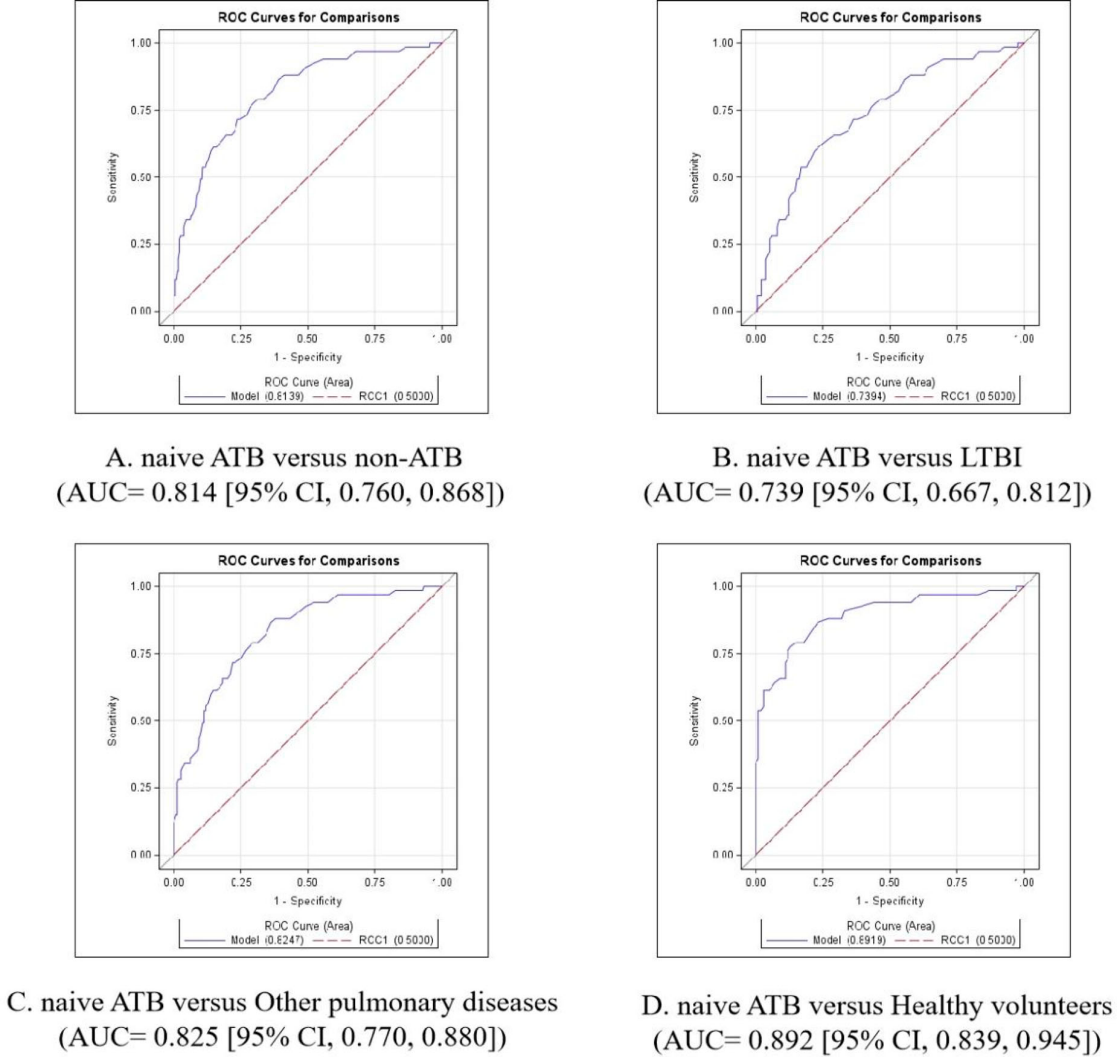
ROC curves for distinguishing naive ATB from non-ATB, latent tuberculosis, other pulmonary diseases, and healthy volunteers.

Next, to further evaluate performance of patients who had not yet started treatment, we evaluated the diagnostic value of the MTB-HR in distinguishing naive ATB from non-ATB, LTBI, other pulmonary diseases, and healthy volunteers. The corresponding AUCs were 0.814 (95% CI, 0.760, 0.868), 0.739 (95% CI, 0.667, 0.812), 0.825 (95% CI, 0.770, 0.880), 0.892 (95% CI, 0.839, 0.945), respectively (Figure 3). For naive ATB versus non-ATB, the cut-point sensitivity was 76.1% (95% CI, 64.7–84.7), and the specificity was 71.6% (95% CI, 67.3–75.5). At a sensitivity of 90%, specificity was 53.4% (95% CI, 48.7–58.0). Pairwise comparisons between naive ATB, LTBI, other pulmonary diseases, and healthy volunteers showed *p* values were less than 0.0001 except for the *p* value of 0.348 in the comparison of other pulmonary diseases and healthy volunteers.

### The performance of MTB-HR in terms of sensitivity and specificity compared to smear, culture, Xpert MTB/RIF and clinical diagnosis of ATB

The appendix described the clinical diagnostic criteria for ATB and the grouping data using the clinical diagnostic criteria. The appendix table showed the performance of MTB-HR in terms of sensitivity and specificity compared to smear, culture, Xpert MTB/RIF and clinical diagnosis of ATB (appendix table). The corresponding AUCs in distinguishing ATB, naive ATB from non-ATB using the clinical diagnostic criteria were 0.774 (95% CI, 0.738–0.811), 0.800 (95% CI, 0.758–0.843), respectively.

The 3-gene TB score cutoff of MTB-HR based on Youden index method is 2.40. Patients with a 3-gene TB score less than 2.40 are categorized as positive, while those with a 3-gene TB score greater than or equal to 2.40 are categorized as negative. Among the 262 patients clinically diagnosed with ATB, the number of positive results were as follows: 167 (MTB-HR), 159 (Xpert MTB/RIF), 149 (culture) and 109 (smear). For 97 cases of naive ATB, the MTB-HR, Xpert MTB/RIF, culture and smear positivity were 70, 51, 55, and 39 respectively.

### Evaluation of 3-gene TB score in participants from different cohorts using TAT grouping

**Figure 4.**
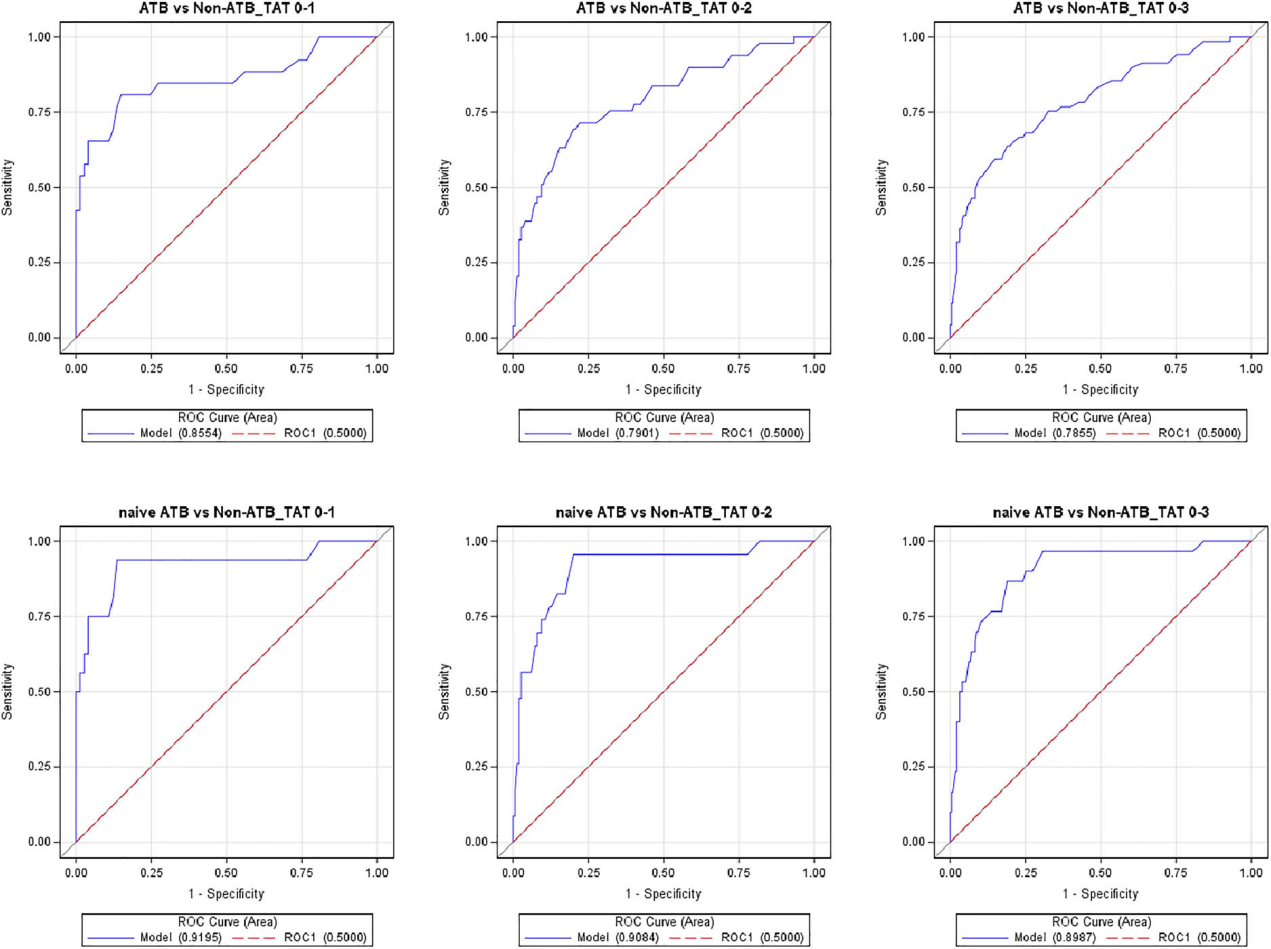
ROC curves for distinguishing participants from different cohorts using TAT grouping.

The diagnostic value of the MTB-HR to distinguish ATB from non-ATB and naive ATB from non-ATB is different using TAT grouping (Figure 4). It is worth noting that only samples with TAT less than 1 h were included (TAT 0-1h), the AUCs of the MTB-HR in distinguishing ATB from non-ATB, naive ATB from non-ATB were largest, 0.855 (95% CI, 0.753-0.957) and 0.920 (95% CI, 0.822–1.000) respectively. For ATB versus non-ATB (TAT 0–1h), the cut-point sensitivity was 76.9% (95% CI, 57.9–89.0), and the specificity was 86.3% (95% CI, 76.6–92.4). For naive ATB versus non-ATB (TAT 0–1h), the cut-point sensitivity was 81.3% (95% CI, 57.0–93.4), and the specificity was 87.7% (95% CI, 78.2–93.4).

### Comparison of 3-gene TB score in participants with ATB against smear, culture time to positivity and IGRA results

In addition, the results of descriptive analysis for the 3-gene TB scores in participants with ATB against smear, Xpert MTB/RIF, culture time to positivity and IGRA results were given in Table 3. The 3-gene TB score of smear 4+ (mean: 0.97) was much lower than that of smear negative (mean 2.03) participants with ATB. Similarly, the 3-gene TB score of ATBs with high semi-quantitative results (mean: 1.06) of GeneXpert MTB/RIF was significantly lower than that of ATBs with negative semi-quantitative results (mean: 2.13). The 3-gene TB scores with a culture time to positivity of less than 14 days (mean: 1.71) was slightly lower than that with a culture time to positivity of more than 28 days (mean: 2.17). In addition, the results showed that the 3-gene TB scores for IGRA negative (mean: 1.53) was slightly lower than that for IGRA positive (mean: 1.84).

**Table 3.** Comparison of 3-gene TB score in participants with ATB against smear, Xpert MTB/RIF, culture time to positivity and IGRA results.

	3-gene TB score	*N*	Minimum	Maximum	Mean	Medium	IQR	Variance	Standard deviation
Smear	Negative	90	−0.15	3.80	2.03	2.20	1.25, 2.80	1.03	1.01
	Scanty	14	0.35	3.05	1.75	1.68	1.300 2.30	0.59	0.77
	1+	40	−0.10	3.75	1.67	1.83	0.80, 2.45	0.99	0.99
	2+	29	−0.95	3.15	1.68	2.00	0.75, 2.60	1.34	1.16
	3+	11	0.00	3.10	1.61	2.00	0.35, 2.75	1.39	1.18
	4+	8	−0.55	2.90	0.97	1.13	−0.38, 1.95	1.73	1.32
GeneXpert MTB/RIF	Negative	33	0.00	3.80	2.10	2.15	1. 50, 2.90	1.06	1.02
	Very low	45	−0.15	3.70	1.72	1.60	1.00, 2.60	1.08	1.04
	Low	49	−0.95	3.75	1.95	2.05	1.50, 2.70	1.19	1.09
	Medium	50	−0.45	3.10	1.81	2.20	1.00, 2.55	1.02	1.01
	High	15	−0.55	2.40	1.06	1.25	0.35, 1.80	0.87	0.93
Culture time to Positivity^a^	0–14d	45	−0.95	3.30	1.71	2.00	0.975, 2.55	1.20	1.10
	14–28d	93	−0.55	3.80	1.89	2.15	0.98, 2.73	1.19	1.09
	>28d	11	0.25	3.55	2.17	2.40	1.45, 2.90	0.97	0.99
IGRA	Negative	17	0.35	3.1	1.53	1.6	0.78, 2.08	0.72	0.85
	Positive	167	−0.95	3.8	1.84	2.05	1.00,2.65	1.15	1.07
	Uncertain	8	0.10	3.75	1.92	1.6	1.33, 3.05	1.37	1.17

^a^ The culture time to positivity refers to the time from the specimen loading to Bactec MGIT 960 instrument to the time when the positive result was reported.

## Discussion

Better diagnostic tools are crucial for detecting TB early and preventing onward transmission. The diagnosis of ATB and LTBI is still a great challenge, relying mainly on traditional smear, culture, molecular biology techniques and IGRA. It had been proven that a three-gene signature (*GBP5*, *DUSP3*, and *KLF2*) is robustly diagnostic for ATB, and has the potential as a clinical tool for monitoring response to TB therapy [4, 9]. The MTB-HR which can quantify relative mRNA levels of the 3-gene signature from a whole-blood sample on the GeneXpert platform is easy to perform and does not require any extensive training of staff. Standard biosafety measurements for blood sampling apply. The total time to obtain result is less than an hour and the required blood sample is only 100uL. Overall, the major advantage of the MTB-HR is in increasing yield compared to current diagnostic methods (e.g. smear, culture, Xpert MTB/RIF and other molecular biological methods) since blood is more regularly available.

We conducted a prospective multi-cohort study of 653 participants in four cohorts to evaluate the performance of the MTB-HR. The results showed that the 3-gene TB scores of the MTB-HR in ATB (mean: 1.815) were lower than those in LTBI (mean: 2.380), other pulmonary diseases (mean: 2.770), and healthy volunteers (mean: 2.934). The diagnostic ability of the MTB-HR to distinguish ATB from LTBI was moderate, with an AUC of 0.660 (sensitivity 49.0%, specificity 76.6%). Whereas, the diagnostic ability of the MTB-HR to distinguish active ATB from healthy volunteers was good, with an AUC of 0.830 (sensitivity 66.1%, specificity 87.0%), slightly lower than the results from the other three studies [6–8]. This may be due to the different population included. This study was conducted in a well-known TB designated hospital, and most of the included ATB were without typical TB symptoms or had received anti-TB treatment. In addition, our study was conducted in China, a country with a high burden of tuberculosis, and it can be a significant limitation when used in countries with a high burden of the disease or among individuals from such countries, where the prevalence of LTBI can be exceptionally high.

Multiple studies have demonstrated that the diagnostic characteristics of ATB based on blood transcriptomics are influenced by anti-TB treatment [10–15]. The study of Zimemer et al demonstrated that the MTB-HR assay detects changes in the 3-gene signature over time with respect to treatment initiation in 31 pulmonary tuberculosis patients [15]. In this study, we found the 3-gene TB scores of the MTB-HR in ATB without anti-TB treatment (mean: 1.530) and those who received 0–8 weeks of treatment (mean: 1.272) were lower than and those who received more than 8 weeks of treatment (mean: 2.178), and were statistically significant (*p* values of less than 0.01). Excluding the influence of tuberculosis treatment, we analyzed the ability of the MTB-HR to distinguish between ATB without anti-TB treatment and non-ATB. The results showed that the AUC for the MTB-HR to distinguish ATB without anti-TB treatment and non-ATB is 0.814 (sensitivity 76.1%, specificity 71.6%), with the highest AUC (0.892, close to 90%, sensitivity 76.1%, specificity 88.0%) seen when differentiating those persons with ATB without anti-TB treatment from healthy volunteers. In this study, the MTB-HR appears more valuable in the diagnosis of untreated ATB.

In addition, it is worth noting that our results showed that there are significant differences in the diagnostic effectiveness of the MTB-HR among different TAT groups. When only samples with TAT less than 1 h were included, the AUCs of the MTB-HR in distinguishing ATB from non-ATB, naive ATB from non-ATB were largest, 0.855 (95% CI, 0.753-0.957,sensitivity 76.9%, specificity 86.3%) and 0.920 (95% CI, 0.822-1.000,sensitivity 81.3%, specificity 87.7%) respectively. This indicates that timely detection after blood sample collection can effectively improve the diagnostic efficacy of the MTB-HR.

Furthermore, we found that the 3-gene TB scores of the MTB-HR in ATB participants with smear 4+ (mean: 0.969), a culture time to positivity of less than 14 days (mean: 1.71), and GeneXpert MTB/RIF high semi-quantitative results (mean:1.060) were much lower than those of smear negative (mean: 2.034), a culture time to positivity of more than 28 days (mean: 2.17), and GeneXpert MTB/RIF negative (mean: 2.127). This suggests that the 3-gene TB scores of the MTB-HR may be related to bacterial load.

Since only 47% of TB in China was microbiological confirmed in 2019[16], we also used clinical diagnostic criteria to classify the participants enrolled in the study, and evaluated the detection ability of the MTB-HR. The results of the AUC in each group were similar to those using CMRS. In addition to the above results, we found that the 3-gene TB scores of the MTB-HR in participants with smear 4+ (mean: 0.969) and GeneXpert MTB/RIF high semi-quantitative results (mean: 1.060) were much lower than those of smear negative (median: 2.034) and GeneXpert MTB/RIF negative (mean: 2.127). This suggests that that the MTB-HR is highly sensitive in detecting individuals with high bacterial load and it is likely that the MTB-HR has low accuracy in diagnosing subclinical TB.

There were several limitations to our study. First, we did not take into account the impact of some potentially interfering factors in the study design phase, such as complications and curative effect. SPH is a tertiary centre and a well-known tuberculosis diagnosis and treatment centre in China, so there are more complicated and critical tuberculosis cases than other hospitals. Considering the above factors, the results of the study may be biased. Second, China had the highest LTBI burden in the world and a population-based study found that IGRA positivity rates ranged from 13% to 20% [17, 18]. It is worth noting that the healthy volunteers in our study did not undergo IGRA testing to exclude LTBI, which may result in LTBI being included in the healthy population. Third, the application of the MTB-HR to clinically diagnosis ATB requires the establishment of a clear threshold value of the 3-gene TB score, which will likely require a multi-centre, multi-population large-scale clinical study. In future studies, we plan to conduct detailed clinical studies to evaluate the effectiveness of the MTB-HR in TB, diagnosis progression, disease severity, and treatment response. At the same time, the influencing factors (including treatment, TAT, etc.) of the MTB-HR testing should be systematically evaluated. It is noteworthy that the diagnostic performance of MTB HR in differentiating ATB from LTBI is not optimal, which may be influenced by multiple factors such as the immune status of the subject. A prospective, large-scale study is on the way to identify related interference factors and optimize the diagnostic algorithm to maximize the diagnostic value.

In conclusion, the MTB-HR assay provides a potential blood-based test for rapid, screening and triage in persons with suspected ATB, especially for untreated ATB. As well, the MTB-HR was highly sensitive in individuals with high bacterial load.

## Supplementary Material

Supplemental Material

## Appendix

According to clinical diagnostic criteria, active TB was defined as either (i) microbiological confirmation of infection with MTB by culture or Xpert MTB/RIF, or (ii) a symptomatic individual fulfilling at least two of the following three criteria in conjunction with response to treatment with anti-tuberculous therapy: (a) symptoms and signs consistent with active TB, (b) radiological findings suggestive of active TB, (c) presence of risk factors for TB infection (known TB contact, birth or previous residence in a high TB prevalence country).

Using clinical diagnostic criteria above, 262 were diagnosed as ATB, and the remaining 391were non-ATB, including 78 cases of LTBI, 213 cases of other pulmonary diseases, and 100 healthy volunteers. According to the anti-TB treatment availability and duration of medication within the last 6 months, 97 patients with ATB were divided into naive ATB without treatment (called “naive ATB”), 41 patients with anti-TB treatment duration from 1 to 8 weeks (called “ATB 0-8w”), and 124patients with anti-TB treatment duration longer than 8 weeks (called “ATB ≥ 8w”).

**Appendix table.** The performance of MTB-HR in terms of sensitivity and specificity compared to smear, culture, Xpert MTB/RIF and clinical diagnosis of ATB.

Description	Cutoff (Youden index)	Sensitivity (95%CI)	Specificity (95%CI)	AUC (95%CI)	Number of subjects
ATB vs Non-ATB (compared to GeneXpert)	1.85	45.3 (38.4, 52.4)	76.8 (69.2, 82.9)	0.659 (0.602, 0.717)	334
ATB vs Non-ATB (compared to Culture)	2.5	68.2 (61.3, 74.4)	59.7 (53.3, 65.9)	0.643 (0.588, 0.698)	423
ATB vs Non-ATB (compared to Smear)	2.5	68.2 (61.3, 74.4)	62.9 (57.6, 68.0)	0.721 (0.673, 0.770)	521
ATB vs Non-ATB (compared to Clinical Diagnosis)	2.4	63.5 (56.5, 70.0)	71.6 (67.3, 75.5)	0.774 (0.738, 0.811)	653
naive ATB vs Non-ATB (compared to GeneXpert)	1.65	53.7 (41.9, 65.1)	81.7 (74.5, 87.2)	0.741 (0.665, 0.816)	209
naive ATB vs Non-ATB (compared to Culture)	2.25	71.6 (59.9, 81.0)	69.3 (63.0, 74.9)	0.760 (0.689, 0.831)	298
naive ATB vs Non-ATB (compared to Smear)	2	61.2 (49.2, 72.0)	80.9 (76.3, 84.7)	0.767 (0.705, 0.829)	396
naive ATB vs Non-ATB (compared to Clinical Diagnosis)	2.4	76.1 (64.7, 84.7)	71.6 (67.3, 75.5)	0.800 (0.758, 0.843)	528
