# Supplementary material for: Assessment of the Cepheid 3-gene Host Response Fingerstick Blood Test (MTB-HR) on rapid diagnosis of tuberculosis
Source: Emerg Microbes Infect. 2023 Oct 17;12(2):2261561. doi: 10.1080/22221751.2023.2261561 (PMC10583623; doi:10.1080/22221751.2023.2261561)
Supplement: Supplementary file 1 [file TEMI_A_2261561_SM2204.docx]

Appendix table The performance of MTB-HR in terms of sensitivity and specificity compared to smear, culture, Xpert MTB/RIF and clinical diagnosis of ATB.

Description	Cutoff (Youden index)	Sensitivity (95%CI)	Specificity (95%CI)	AUC (95%CI)	Number of subjects
ATB vs Non-ATB (compared to GeneXpert)	1.85	45.3 (38.4, 52.4)	76.8 (69.2, 82.9)	0.659 (0.602, 0.717)	334
ATB vs Non-ATB (compared to Culture)	2.5	68.2 (61.3, 74.4)	59.7 (53.3, 65.9)	0.643 (0.588, 0.698)	423
ATB vs Non-ATB (compared to Smear)	2.5	68.2 (61.3, 74.4)	62.9 (57.6, 68.0)	0.721 (0.673, 0.770)	521
ATB vs Non-ATB (compared to Clinical Diagnosis)	2.4	63.5 (56.5, 70.0)	71.6 (67.3, 75.5)	0.774 (0.738, 0.811)	653
naive ATB vs Non-ATB (compared to GeneXpert)	1.65	53.7 (41.9, 65.1)	81.7 (74.5, 87.2)	0.741 (0.665, 0.816)	209
naive ATB vs Non-ATB (compared to Culture)	2.25	71.6 (59.9, 81.0)	69.3 (63.0, 74.9)	0.760 (0.689, 0.831)	298
naive ATB vs Non-ATB (compared to Smear)	2	61.2 (49.2, 72.0)	80.9 (76.3, 84.7)	0.767 (0.705, 0.829)	396
naive ATB vs Non-ATB (compared to Clinical Diagnosis)	2.4	76.1 (64.7, 84.7)	71.6 (67.3, 75.5)	0.800 (0.758, 0.843)	528
